# Supplementary material for: Effect of music on preoperative anxiety and postoperative pain in patients undergoing gynecological surgery: A meta-analysis
Source: PLoS One. 2025 Apr 29;20(4):e0319639. doi: 10.1371/journal.pone.0319639 (PMC12040270; doi:10.1371/journal.pone.0319639)
Supplement: Attachment — Submitted filename: Response to Reviewers.docx [file pone.0319639.s006.docx]

**Comments to the Author**

2. We note that your Data Availability Statement is currently as follows: [All relevant data are within the manuscript and its Supporting Information files.]

Please confirm at this time whether or not your submission contains all raw data required to replicate the results of your study. Authors must share the “minimal data set” for their submission. PLOS defines the minimal data set to consist of the data required to replicate all study findings reported in the article, as well as related metadata and methods ([https://journals.plos.org/plosone/s/data-availability#loc-minimal-data-set-definition](https://journals.plos.org/plosone/s/data-availability#loc-minimal-data-set-definition)).

For example, authors should submit the following data:

- The values behind the means, standard deviations and other measures reported;
- The values used to build graphs;
- The points extracted from images for analysis.

Answer:All relevant data are within the manuscript.

5. Review Comments to the Author

Please use the space provided to explain your answers to the questions above. You may also include additional comments for the author, including concerns about dual publication, research ethics, or publication ethics. (Please upload your review as an attachment if it exceeds 20,000 characters)

Reviewer #1: Lin et al. present a very interesting study titled “Effect of Music on preoperative anxiety and postoperative pain in patients undergoing gynecological surgery”. This study shows that musical intervention was effective in reducing anxiety and pain in patients undergoing gynaecological surgery. However, to improve the quality of their manuscript I would recommend the authors make the following edits:
1. Background Section, Page 8: The rationale should focus more specifically on music intervention by highlighting its unique benefits. The authors could do so by citing studies or providing evidence demonstrating the effectiveness of music intervention in reducing preoperative anxiety and improving patient outcomes, especially when compared to alternative non-pharmacological approaches.
2. Background Section, Page 8: The background section should focus more on gynaecological surgery, aligning with the research topic. The authors should highlight how studying musical intervention can impact preoperative anxiety in this patient population specifically as this can significantly enhance the study's relevance. Moreover, the authors could talk about how anxiety is notably prevalent in gynaecological operations and how addressing this anxiety can impact surgical outcomes.

Answer:

Background

Preoperative anxiety is mainly manifested as worries and fears about the surgery, such as tachycardia, arrhythmia, or hypertension. [1,2]It is understandable for patients to experience mild anxiety before and after surgery, as it can enhance their adaptation to the external environment. However, severe anxiety can often interfere with the process of recovery, and be considered a mental disorder, and a disease. [3]

According to reports, the overall prevalence of preoperative anxiety among 14,000 surgical patients worldwide is 48%. During the waiting time before surgery, anxiety symptoms worsen in 80% of surgical patients. Common fears of surgical patients include surgical complications, the duration and degree of disability after surgery, general anesthesia and related loss of control, and waking up during or after surgery and experiencing discomfort and pain. [4, 5]

This is particularly true for gynecologic surgery, which often results in the partial or total removal of organs associated with fertility and female characteristics. Sexual desire may be impaired [6]. For example, the physical and psychological effects after hysterectomy are significant, including hot flashes, night sweats, urinary incontinence, and urge incontinence [7]. Patients have reported fears of pain, feelings of rejection by their spouses, and sensations of dismemberment [8]. The surgical treatment of gynecologic cancer can have both short- and long-term effects on sexual health, emotional well-being, reproductive function, and overall quality of life [9].

Preoperative anxiety may lead to increased surgical risks, higher anesthesia requirements, and more postoperative complications, including heightened postoperative pain, nausea, vomiting, and prolonged hospital or postoperative stay. [10, 11]

In addition, preoperative anxiety symptoms also serve as predictors of reduced satisfaction and are subjective indicators of treatment efficacy from the patient's viewpoint. [12,13] Particular note is that an increase in preoperative anxiety is also a risk factor for postoperative mortality. [14-16]

There are different treatment options for preoperative anxiety, including both pharmacological and non-pharmacological methods. Non-pharmacological interventions are becoming increasingly popular due to the potential adverse effects of pharmacological treatment, such as respiratory problems, drowsiness, interference with anesthetic medication, and prolonged recovery time. These methods include cognitive-behavioral therapy, music therapy, preoperative preparation videos, aromatherapy, hypnosis, guided imagery relaxation therapy, and massage. [17]

In conclusion, music intervention may provide a viable alternative to sedatives and anti-anxiety medications to reduce preoperative anxiety in surgical procedures. [18]

In surgical procedures, such as adenocarcinoma surgery in women, interventions like music, aromatherapy, massage, and virtual reality have been shown to reduce preoperative anxiety and postoperative pain. These interventions demonstrate cost-effectiveness, minimal invasiveness, and a low risk of side effects. However, this study does not determine which of these measures is the most effective. Music, in particular, does not require significant manpower or material resources, incurs lower costs, and is therefore recommended as the most favorable intervention. [19-21]

However, there is no relevant systematic review or meta-analysis to evaluate whether anxiety symptoms in patients before gynecological surgery can be improved through music intervention. Therefore, we searched for relevant studies to evaluate whether music intervention can alleviate preoperative anxiety and postoperative pain.

3. Methods Section, Page 9: The inclusion criteria states that studies with “females undergoing gynecological surgery," were included, this is too broad and vague. Please specify the types of gynecological surgeries performed in the included studies. If the studies encompassed a range of surgeries, it would be beneficial to mention these in the baseline characteristics table.

Answer:

2.1 Inclusion and Exclusion Criteria

Inclusion criteria:(1) inclusion of randomized controlled trials (RCTs), observational studies, comparative studies, clinical trials, clinical studies, multicenter studies, and case-control studies; (2) female patients undergoing gynecological surgery; including partial hysterectomy,dilatation and curettage, completion curettage, tubal ligation, total hysterectomy, myomectomy, pelvic reconstructive surgery and so on.(3) all experimenters undergo music intervention before and after surgery; (4) outcome measures include preoperative anxiety and postoperative pain. (5) Literature utilizing the State-Trait Anxiety Inventory Y Form (STAI-Y) to assess anxiety. (6) Literature is limited to English only.

4. Results Section, Page 13: The authors have discussed "preoperative anxiety before and after listening to music" in section 3.3.3, which is supposed to address postoperative pain. Please correct this mistake to ensure accuracy and clarity in the manuscript.

Answer:3.3.3 Meta-analysis comparing pain in patients who listened to music and control patients after surgery

This meta-analysis included three studies. Two studies were randomized controlled trials with a risk of bias, so the quality of evidence for each study was downgraded by one level. Another study was a case-control study with a large effect size and no significant confounding factors, so the quality of evidence was upgraded by one level. Therefore, the level of evidence quality for comparing postoperative pain after patients listen to music was moderate. This results of the evidence quality assessment of the studies are shown in Supplementary Table 3.

5. Discussion Section, Pages 20-21: The discussion section should explicitly address the medical importance and practical implications of the study's results. It is essential to explain exactly how these findings can enhance patient care in clinical settings, especially in gynaecological surgery.
6. Discussion Section, Page 20: The authors have provided a very brief explanation of how music alleviates anxiety and pain, which is insufficient and lacks clarity. Therefore, I would recommend that they expand on this topic by offering a more thorough, specific, and clear explanation of the mechanisms through which music influences anxiety and pain. This could involve providing specific explanations of the physiological processes or other mechanisms supported by the current literature.
7. There are several grammar and typographical issues in the manuscript, that the authors need to address. For instance, in the discussion section, paragraphs vary in spacing, with some double-spaced and others lacking proper spacing. Additionally, different fonts are used sporadically, and there is inconsistent spacing between paragraphs and sections throughout the manuscript. The presentation and formatting of the tables need improvement as insufficient spacing between rows and columns makes it challenging to discern headings and sections clearly. Moreover, the reference list contains unnumbered references, which affects the overall organization and readability of citations.

Answer:

4. Discussion

In this paper, we analyzed the effects of music on preoperative anxiety and postoperative pain in patients in gynecological surgery. This meta-analysis indicates that patients who listened to music before surgery had significantly improved preoperative anxiety compared to the control group.This meta-analysis also indicates that patients who listened to music after surgery had significant relief in postoperative pain. Moreover, there was a significant improvement in anxiety symptoms after listening to music compared to before. In summary, we should listen to music for patients before and after gynecological surgery.Listening to music can alleviate preoperative anxiety and postoperative pain in patients undergoing gynecological surgery.

Music is one of the most commonly used non-pharmacological interventions to reduce anxiety. Music is a way for people to relax.[31] It helps patients overcome emotional and physical alienation, providing comfort and familiarity in an improved environment, and serves as a pleasant distraction from pain and anxiety. [32]Music significantly reduces preoperative patients' anxiety, heart rate, and other vital signs. [33]

We analyzed the mechanism by which music affects patient anxiety.Music is a powerful stimulus that evokes and regulates emotions[34]. Studies have shown that the subject's anxiety may be associated with amygdala activity, and music has a significant impact on the amygdala. [36, 37]Music can also have a direct impact on the limbic system of the brain, the reticular structure of the brainstem, and the body's internal organs and functions. For people, harmonious melodies can enhance mental well-being, divert attention, promote relaxation, and reduce tension and anxiety.[38]

Regarding the effect of music on anxiety, other studies have also confirmed the findings of this meta-analysis. In a study on anxiety during cesarean section, researchers found that the anxiety levels of patients in the music group during surgery were lower than those of the control group. [31]It was consistent with the results of our study.

In a study on music intervention for dental anxiety patients, it was found that music significantly reduced heart rate, systolic blood pressure fluctuation, diastolic blood pressure fluctuation, anxiety scores, pain scores. Music also significantly decreased anxiety and pain levels while improving patient cooperation. [39]It was consistent with the results of our study.

In a study on music intervention for cataract surgery patients, researchers found that listening to music during cataract surgery significantly reduced self-reported anxiety levels before, during, and after the procedure. Moreover, it led to a significant decrease in postoperative blood pressure. [40]

Another study also indicated that listening to music is associated with a significant reduction in anxiety and pain after cardiac surgery. [41]It was consistent with the results of our study.

Because of the risk of bias in the randomized controlled trials and the inclusion of observational studies in this paper, the quality of evidence was rated as low to medium. This indicates that there is limited confidence in these results, and the actual outcomes may differ from those reported. Therefore, more and better quality randomized controlled trials and adherence to reporting bias control systems are recommended.

The conclusions of these studies are consistent with the findings of this meta-analysis, indicating that music can alleviate preoperative anxiety and postoperative pain in patients. It's totally confirmed that music is an effective, safe, and inexpensive non-pharmacological intervention. It can reduce preoperative anxiety and postoperative pain in patients. This is a good choice for preoperative patients to listen to music before and after surgery. It also plays a supportive role for operating room nurses in patient care.

References

[1] Hu JY, Zhou QM, Li WJ, Deng XL, Wang WY, Huang RL, Huang JW. Effects of Preoperative Anxiety Status on Haemodynamic Changes during and after Tooth Extraction in Middle-aged and Elderly Patients with Hypertension: a Prospective Repeated-Measures Cohort Study. Chin J Dent Res. 2021 Dec 23;24(4):267-274.

[2]Tully PJ, Bennetts JS, Baker RA, McGavigan AD, Turnbull DA, Winefield HR. Anxiety, depression, and stress as risk factors for atrial fibrillation after cardiac surgery. Heart Lung. 2011 Jan-Feb;40(1):4-11.

[3]Andersson V, Bergstrand J, Engström Å, Gustafsson S. The Impact of Preoperative Patient Anxiety on Postoperative Anxiety and Quality of Recovery After Orthopaedic Surgery. J Perianesth Nurs. 2020 Jun;35(3):260-264.

[4] Bansal T, Joon A. Preoperative anxiety-an important but neglected issue: A narrative review. The Indian Anaesthetists’ Forum. 2016; 17(2): 37.

[5] Friedrich S, Reis S, Meybohm P, Kranke P. Preoperative anxiety. Curr Opin Anaesthesiol. 2022 Dec 1;35(6):674-678.

[6] Pauls RN. Impact of gynecological surgery on female sexual function. Int J Impot Res. avr. 2010;22(2):105–114.

[7] Kołodziejczyk A, Pawłowski T. Negative body image in breast cancer patients. Adv Clin Exp Med Off Organ Wroclaw Med Univ. août. 2019;28(8):1137–1142.

[8]Gueye M, Diouf AA, Cisse A, Coulbary AS, Moreau JC, Diouf A. Consequences of hysterectomy at the national-hospital of Pikine in Dakar.

[9] Stabile C, Gunn A, Sonoda Y, Carter J. Emotional and sexual concerns in women undergoing pelvic surgery and associated treatment for gynecologic cancer. Transl Androl Urol. 2015;4(2):169–185.

[10] Celik F, Edipoglu IS. Evaluation of preoperative anxiety and fear of anesthesia using APAIS score. Eur J Med Res. 2018 Sep 11;23(1):41.

[11]Jaruzel CB, Gregoski MJ. Instruments to Measure Preoperative Acute Situational Anxiety: An Integrative Review. AANA J. 2017 Feb;85(1):31-35.

[12] Ali A, Lindstrand A, Sundberg M, Flivik G. Preoperative Anxiety and Depression Correlate With Dissatisfaction After Total Knee Arthroplasty: A Prospective Longitudinal Cohort Study of 186 Patients, With 4-Year Follow-Up. J Arthroplasty. 2017 Mar;32(3):767-770.

[13] González-Lemonnier S, Bovaira-Forner M, Peñarrocha-Diago M, Peñarrocha-Oltra D. Relationship between preoperative anxiety and postoperative satisfaction in dental implant surgery with intravenous conscious sedation. Med Oral Patol Oral Cir Bucal. 2010 Mar 1;15(2):e379-82.

[14] Cserép Z, Losoncz E, Balog P, Szili-Török T, Husz A, Juhász B, Kertai MD, Gál J, Székely A. The impact of preoperative anxiety and education level on long-term mortality after cardiac surgery. J Cardiothorac Surg. 2012 Sep 14;7:86.

[15] Székely A, Balog P, Benkö E, Breuer T, Székely J, Kertai MD, Horkay F, Kopp MS, Thayer JF. Anxiety predicts mortality and morbidity after coronary artery and valve surgery--a 4-year follow-up study. Psychosom Med. 2007 Sep-Oct;69(7):625-31. doi: 10.1097/PSY.0b013e31814b8c0f. Epub 2007 Aug 27.

[16] Williams JB, Alexander KP, Morin JF, Langlois Y, Noiseux N, Perrault LP, Smolderen K, Arnold SV, Eisenberg MJ, Pilote L, Monette J, Bergman H, Smith PK, Afilalo J. Preoperative anxiety as a predictor of mortality and major morbidity in patients aged >70 years undergoing cardiac surgery. Am J Cardiol. 2013 Jan 1;111(1):137-42.

[17]Wang R, Huang X, Wang Y, Akbari M. Non-pharmacologic Approaches in Preoperative Anxiety, a Comprehensive Review. Front Public Health. 2022 Apr 11;10:854673.

[18]Bradt J, Dileo C, Shim M. Music interventions for preoperative anxiety. Cochrane Database Syst Rev. 2013 Jun 6;2013(6):CD006908.

[19]Agüero-Millan B, Abajas-Bustillo R, Ortego-Maté C. Efficacy of nonpharmacologic interventions in preoperative anxiety: A systematic review of systematic reviews. J Clin Nurs. 2023 Sep;32(17-18):6229-6242.

[20]Guo P, Li P, Zhang X, Liu N, Wang J, Yang S, Yu L, Zhang W. The effectiveness of aromatherapy on preoperative anxiety in adults: A systematic review and meta-analysis of randomized controlled trials. Int J Nurs Stud. 2020 Nov;111:103747.

[21]Tola YO, Chow KM, Liang W. Effects of non-pharmacological interventions on preoperative anxiety and postoperative pain in patients undergoing breast cancer surgery: A systematic review. J Clin Nurs. 2021 Dec;30(23-24):3369-3384.

[22] Luchini C SB, Solmi M, Veronese N. Assessing the quality of studies in meta-analyses: advantages and limitations of the Newcastle Ottawa Scale. World J Meta-Anal. (2017) 5:80–4.

[23] Jadad AR, Moore RA, Carroll D, Jenkinson C, Reynolds DJ, Gavaghan DJ, McQuay HJ. Assessing the quality of reports of randomized clinical trials: is blinding necessary? Control Clin Trials. 1996 Feb;17(1):1-12.

[24] Balshem H, Helfand M, Schünemann HJ, Oxman AD, Kunz R, Brozek J, Vist GE, Falck-Ytter Y, Meerpohl J, Norris S, Guyatt GH. GRADE guidelines: 3. Rating the quality of evidence. J Clin Epidemiol. 2011 Apr;64(4):401-6.

[25] Guyatt GH, Oxman AD, Vist GE, Kunz R, Falck-Ytter Y, Alonso-Coello P, Schünemann HJ; GRADE Working Group. GRADE: an emerging consensus on rating quality of evidence and strength of recommendations. BMJ. 2008 Apr 26;336(7650):924-6.

[26] Labrague LJ, McEnroe-Petitte DM. Influence of Music on Preoperative Anxiety and Physiologic Parameters in Women Undergoing Gynecologic Surgery. Clin Nurs Res. 2016 Apr;25(2):157-73.

[27] Casarin J, Cromi A, Sgobbi B, Di Siena A, Serati M, Bolis ME, Ghezzi F. Music Therapy for Preoperative Anxiety Reduction in Women Undergoing Total Laparoscopic Hysterectomy: A Randomized Controlled Trial. J Minim Invasive Gynecol. 2021 Sep;28(9):1618-1624.e1.

[28] Good M, Ahn S. Korean and American music reduces pain in Korean women after gynecologic surgery. Pain Manag Nurs. 2008 Sep;9(3):96-103.

[29] Chen YB, Barnes H, Westbay L, Wolff B, Shannon M, Adams W, Acevedo-Alvarez M, Mueller ER, Pham TT. Preoperative Music Listening in Pelvic Reconstructive Surgery: A Randomized Trial. Female Pelvic Med Reconstr Surg. 2021 Aug 1;27(8):469-473.

[30] Choi EK, Baek J, Lee D, Kim DY. Effect on music therapy on quality of recovery and postoperative pain after gynecological laparoscopy. Medicine (Baltimore). 2023 Mar 3;102(9):e33071.

[31] Schäfer T, Sedlmeier P, Städtler C, Huron D. The psychological functions of music listening. Front Psychol. 2013 Aug 13;4:511.

[32] Fenko A, Loock C. The influence of ambient scent and music on patients' anxiety in a waiting room of a plastic surgeon. HERD. 2014 Spring;7(3):38-59.

[33] Augustin P, Hains AA. Effect of music on ambulatory surgery patients' preoperative anxiety. AORN J. 1996 Apr;63(4):750, 753-8.

[34]Aalbers S, Fusar-Poli L, Freeman RE, Spreen M, Ket JC, Vink AC, Maratos A, Crawford M, Chen XJ, Gold C. Music therapy for depression. Cochrane Database Syst Rev. 2017 Nov 16;11(11):CD004517.

[35] Etkin A, Wager TD. Functional neuroimaging of anxiety: a meta-analysis of emotional processing in PTSD, social anxiety disorder, and specific phobia. Am J Psychiatry. 2007 Oct;164(10):1476-88.

[36] Koelsch S, Fritz T, V Cramon DY, Müller K, Friederici AD. Investigating emotion with music: an fMRI study. Hum Brain Mapp. 2006 Mar;27(3):239-50.

[37] Weingarten SJ, Levy AT, Berghella V. The effect of music on anxiety in women undergoing cesarean delivery: a systematic review and meta-analysis. Am J Obstet Gynecol MFM. 2021 Sep;3(5):100435.

[38]Liu S, Li G. Analysis of the Effect of Music Therapy Interventions on College Students with Excessive Anxiety. Occup Ther Int. 2023 Mar 24;2023:3351918.

[39] Tan K, Liu H, Huang S, Li C. Efficacy of Music Intervention for Dental Anxiety Disorders: A Systematic Review and Meta-Analysis. Medicina (Kaunas). 2023 Jan 20;59(2):209.

[40] Muddana SK, Hess OM, Sundar S, Venkatesh R. Preoperative and perioperative music to reduce anxiety during first-time phacoemulsification cataract surgery in the high-volume setting: randomized controlled trial. J Cataract Refract Surg. 2021 Apr 1;47(4):471-475.

[41] Kakar E, Billar RJ, van Rosmalen J, Klimek M, Takkenberg JJM, Jeekel J. Music intervention to relieve anxiety and pain in adults undergoing cardiac surgery: a systematic review and meta-analysis. Open Heart. 2021 Jan;8(1):e001474.

Reviewer #2: 1. The introduction should be divided into three parts and provide a strong rationale. Additionally, the flow should be improved for better consistency.

2. The inclusion and exclusion criteria paragraph should be mentioned after the search strategy paragraph.

Answer:2.1 Inclusion and Exclusion Criteria

Inclusion criteria:(1) inclusion of randomized controlled trials (RCTs), observational studies, comparative studies, clinical trials, clinical studies, multicenter studies, and case-control studies; (2) female patients undergoing gynecological surgery; including partial hysterectomy,dilatation and curettage, completion curettage, tubal ligation, total hysterectomy, myomectomy, pelvic reconstructive surgery and so on.(3) all experimenters undergo music intervention before and after surgery; (4) outcome measures include preoperative anxiety and postoperative pain. (5) Literature utilizing the State-Trait Anxiety Inventory Y Form (STAI-Y) to assess anxiety. (6) Literature is limited to English only.

3. Ethical considerations(2.8) should be addressed in the declarations section following the discussion, rather than in the methodology. If they are already included in the declarations section, they should not be repeated.

Answer: have deleted

4. Review the use of abbreviations throughout your article to ensure consistency and avoid repetition.

5. The meta-analysis should be conducted according to PRISMA guidelines, but this study has not mentioned adherence to these guidelines.

6. The author should provide a detailed search strategy as a supplementary file.

Answer: 2.2 Search strategy

To identify studies that meet the inclusion criteria, computer searches and manual searches were conducted in the PubMed, Embase, Web of Science, and Cochrane Library databases from the date of database establishment to April 2024. The search was limited to English-language publications and was not restricted by the year of publication. Search terms included "music" and "gynecological”or “preoperative anxiety” or “postoperative pain”. Two researchers independently conducted computer searches and manual searches of literature, and a third researcher was consulted if there was a disagreement in opinions.

7. Headings of paragraphs should be in bold letters, and full words should be used, such as "discussion" instead of "discuss."

Answer: 5.Discussion

In this paper, we analyzed the effects of music on preoperative anxiety and postoperative pain in patients in gynecological surgery. This meta-analysis indicates that patients who listened to music before surgery had significantly improved preoperative anxiety compared to the control group.This meta-analysis also indicates that patients who listened to music after surgery had significant relief in postoperative pain. Moreover, there was a significant improvement in anxiety symptoms after listening to music compared to before. In summary, we should listen to music for patients before and after gynecological surgery.Listening to music can alleviate preoperative anxiety and postoperative pain in patients undergoing gynecological surgery.

8. The fonts are not consistent throughout the manuscript, and there are irregular spaces between the lines which shows lack of professionalism.

9. The discussion section is unsatisfactory; the flow of paragraphs lacks proper continuity. The discussion should begin by stating the findings, followed by an explanation of various studies.

Answer: 5.Discussion

In this paper, we analyzed the effects of music on preoperative anxiety and postoperative pain in patients in gynecological surgery. This meta-analysis indicates that patients who listened to music before surgery had significantly improved preoperative anxiety compared to the control group.This meta-analysis also indicates that patients who listened to music after surgery had significant relief in postoperative pain. Moreover, there was a significant improvement in anxiety symptoms after listening to music compared to before. In summary, we should listen to music for patients before and after gynecological surgery.Listening to music can alleviate preoperative anxiety and postoperative pain in patients undergoing gynecological surgery.

Music is one of the most commonly used non-pharmacological interventions to reduce anxiety. Music is a way for people to relax.[31] It helps patients overcome emotional and physical alienation, providing comfort and familiarity in an improved environment, and serves as a pleasant distraction from pain and anxiety. [32]Music significantly reduces preoperative patients' anxiety, heart rate, and other vital signs. [33]

We analyzed the mechanism by which music affects patient anxiety.Music is a powerful stimulus that evokes and regulates emotions[34]. Studies have shown that the subject's anxiety may be associated with amygdala activity, and music has a significant impact on the amygdala. [36, 37]Music can also have a direct impact on the limbic system of the brain, the reticular structure of the brainstem, and the body's internal organs and functions. For people, harmonious melodies can enhance mental well-being, divert attention, promote relaxation, and reduce tension and anxiety.[38]

Regarding the effect of music on anxiety, other studies have also confirmed the findings of this meta-analysis. In a study on anxiety during cesarean section, researchers found that the anxiety levels of patients in the music group during surgery were lower than those of the control group. [31]It was consistent with the results of our study.

In a study on music intervention for dental anxiety patients, it was found that music significantly reduced heart rate, systolic blood pressure fluctuation, diastolic blood pressure fluctuation, anxiety scores, pain scores. Music also significantly decreased anxiety and pain levels while improving patient cooperation. [39]It was consistent with the results of our study.

In a study on music intervention for cataract surgery patients, researchers found that listening to music during cataract surgery significantly reduced self-reported anxiety levels before, during, and after the procedure. Moreover, it led to a significant decrease in postoperative blood pressure. [40]

Another study also indicated that listening to music is associated with a significant reduction in anxiety and pain after cardiac surgery. [41]It was consistent with the results of our study.

Because of the risk of bias in the randomized controlled trials and the inclusion of observational studies in this paper, the quality of evidence was rated as low to medium. This indicates that there is limited confidence in these results, and the actual outcomes may differ from those reported. Therefore, more and better quality randomized controlled trials and adherence to reporting bias control systems are recommended.

The conclusions of these studies are consistent with the findings of this meta-analysis, indicating that music can alleviate preoperative anxiety and postoperative pain in patients. It's totally confirmed that music is an effective, safe, and inexpensive non-pharmacological intervention. It can reduce preoperative anxiety and postoperative pain in patients. This is a good choice for preoperative patients to listen to music before and after surgery. It also plays a supportive role for operating room nurses in patient care.

10. Abbreviations should not be placed at the end of the manuscript. Essential abbreviations should be defined at their first mention in the text.

Answer: have deleted

11. The results of the evidence quality assessment of the studies should not be included in the main manuscript text. Instead, they should be provided as supplementary materials, along with the original images from the ROB tool.

6. PLOS authors have the option to publish the peer review history of their article ([what does this mean?](https://c05y1x9s.r.us-east-2.awstrack.me/L0/https://journals.plos.org/plosone/s/editorial-and-peer-review-process%23loc-peer-review-history/1/010f0191756ce17b-b0ba576f-2eca-4656-85ee-291ed34fb2b0-000000/R0IYkW3AOa7PSgNHycfhn1GRzxM=173)). If published, this will include your full peer review and any attached files.
