# Supplementary material for: Effect of music on preoperative anxiety and postoperative pain in patients undergoing gynecological surgery: A meta-analysis
Source: PLoS One. 2025 Apr 29;20(4):e0319639. doi: 10.1371/journal.pone.0319639 (PMC12040270; doi:10.1371/journal.pone.0319639)
Supplement: Attachment — Submitted filename: Response_to_Reviewers_auresp_2.docx [file pone.0319639.s007.docx]

6. Review Comments to the Author

Please use the space provided to explain your answers to the questions above. You may also include additional comments for the author, including concerns about dual publication, research ethics, or publication ethics. (Please upload your review as an attachment if it exceeds 20,000 characters)

Reviewer #1: The authors have addressed the majority of the comments. However, my last comment regarding the formatting and presentation of the tables has not been fully addressed. The tables are still difficult to read due to issues with their formatting, spacing, and overall presentation. I recommend that the authors revise the tables to improve clarity and ensure that the data is easily interpretable.

However, my last comment regarding the formatting and presentation of the tables has not been fully addressed. The tables are still difficult to read due to issues with their formatting, spacing, and overall presentation.

Revise:

| Study | Study type | Intervening measure | Age (years) | | Sample | Time of  collection | Outcome |
| --- | --- | --- | --- | --- | --- | --- | --- |
|  |  |  | Experimental group | Control group | Experimental group/Control group |  |  |
| Leodoro J  Labrague  2016 | case-control trial | music (before surgery) | 42.31 ±  4.89 | 42.10 ±  5.02 | 48/49 | before surgery | preoperative anxiety |
| [Jvan Casarin 2021](https://pubmed.ncbi.nlm.nih.gov/?size=200&term=Casarin+J&cauthor_id=33549732) | RCT | music (before and after  surgery) | 46.6−49.8 | 49.4−51.7 | 30/70 | Before / 6  hours after  surgery | preoperative anxiety, and  postoperative pain |
| Marion Good 2008 | case-control trial | music (after surgery) | 37±7 | 46± 9 | 34/39 | the second  day after  surgery | postoperative pain |
| Eun Kyung Choi 2023 | RCT | music (after surgery) | 46.4 ± 11.8 | 48.3 ± 13.8 | 41/41 | 36 hours  after surgery | postoperative pain |
| [Yufan Brandon Chen 2021](https://pubmed.ncbi.nlm.nih.gov/?size=200&term=Chen+YB&cauthor_id=34397606) | RCT | music (before surgery) | 60.2 ±12.3 | 60.8 ±12.5 | 32/34 | before surgery | preoperative anxiety |

| Table 2A NOS | | | | | | | | | |
| --- | --- | --- | --- | --- | --- | --- | --- | --- | --- |
| Study | I | II | III | IV | V | VI | VII | VIII | Total points |
| Leodoro J Labrague 2016 | 1 | 0 | 1 | 1 | 1 | 1 | 1 | 0 | 6 |
| Marion Good 2008 | 1 | 0 | 1 | 1 | 1 | 1 | 1 | 0 | 6 |
| Note:I Case identif-ication is appropriate. II Repres-entative of cases. III The selection of controls. IV Determ-ination of contrast. V The comparability of cases and controls was considered for the design and statistical analysis. VI Determi-nation of the exposure factors VII The same method was used to determine the exposure factors in the cases and controls. VIII No response rate. | | | | | | | | | |

| Table 2B Jadad score | | | | | |
| --- | --- | --- | --- | --- | --- |
| Study | Generation of the random sequences | Randomization hidden | blind method | Withdrawal and loss of follow-up | total points |
| [Jvan Casarin 2021](https://pubmed.ncbi.nlm.nih.gov/?size=200&term=Casarin+J&cauthor_id=33549732) | 2 | 2 | 0 | 0 | 4 |
| Eun Kyung Choi 2023 | 2 | 1 | 2 | 0 | 5 |
| Yufan Brandon Chen 2021 | 2 | 2 | 0 | 1 | 5 |

Table 2 Quality scores of the studies
