# Supplementary material for: Effect of music on preoperative anxiety and postoperative pain in patients undergoing gynecological surgery: A meta-analysis
Source: PLoS One. 2025 Apr 29;20(4):e0319639. doi: 10.1371/journal.pone.0319639 (PMC12040270; doi:10.1371/journal.pone.0319639)
Supplement: Attachment — Submitted filename: Response_to_Reviewers_auresp_3.docx [file pone.0319639.s008.docx]

Response to Reviewers

6. Review Comments to the Author

Please use the space provided to explain your answers to the questions above. You may also include additional comments for the author, including concerns about dual publication, research ethics, or publication ethics. (Please upload your review as an attachment if it exceeds 20,000 characters)

Reviewer #2: Paraphrase grammar errors and proofread your manuscript. Carefully review your text for errors in spelling, grammar and consistency or sentence flow.

Reviewer #3: The introduction gives a good overview of preoperative anxiety and how it affects patients, especially in gynecologic surgery. However, it would help to mention the main goal of your study earlier. This way, readers know what to expect right from the start.

In introduction, Some sentences are long and can be broken down into shorter ones for better readability. Try to make your points clear and concise.

Revise:**Background**

Preoperative anxiety is mainly manifested as worries and fears about the surgery, such as tachycardia, arrhythmia, or hypertension. [1,2]It is understandable for patients to experience mild anxiety before and after surgery, as it can enhance their adaptation to the external environment. However, severe anxiety can often interfere with the process of recovery, and be considered a mental disorder, and a disease. [3]

According to reports, the overall prevalence of preoperative anxiety among 14,000 surgical patients worldwide is 48%. During the waiting time before surgery, anxiety symptoms worsen in 80% of surgical patients. Common fears of surgical patients include surgical complications, the duration and degree of disability after surgery, general anesthesia and related loss of control, and waking up during or after surgery and experiencing discomfort and pain. [4, 5]

This is particularly true for gynecologic surgery, which often results in the partial or total removal of organs associated with fertility and female characteristics. Sexual desire may be impaired [6]. For example, the physical and psychological effects after hysterectomy are significant, including hot flashes, night sweats, urinary incontinence, and urge incontinence [7]. Patients have reported fears of pain, feelings of rejection by their spouses, and sensations of dismemberment [8]. The surgical treatment of gynecologic cancer can have both short- and long-term effects on sexual health, emotional well-being, reproductive function, and overall quality of life [9]. Therefore, it is necessary to study the anxiety and pain of patients before and after gynecological surgery.

Preoperative anxiety may lead to increased surgical risks, higher anesthesia requirements. And It also may lead more postoperative complications, including heightened postoperative pain, nausea, vomiting, and prolonged hospital or postoperative stay. [10, 11]

In addition, preoperative anxiety symptoms also serve as predictors of reduced satisfaction. And preoperative anxiety symptoms are subjective indicators of treatment efficacy from the patient's viewpoint. [12,13] Particular note is that an increase in preoperative anxiety is also a risk factor for postoperative mortality. [14-16]

There are different treatment options for preoperative anxiety, including both pharmacological and non-pharmacological methods. Non-pharmacological interventions are becoming increasingly popular due to the potential adverse effects of pharmacological treatment, such as respiratory problems, drowsiness, interference with anesthetic medication, and prolonged recovery time. These methods include cognitive-behavioral therapy, music therapy, preoperative preparation videos, aromatherapy, hypnosis, guided imagery relaxation therapy, and massage. [17]In conclusion, music intervention may provide a viable alternative to sedatives and anti-anxiety medications to reduce preoperative anxiety in surgical procedures. [18]

In surgical procedures, such as adenocarcinoma surgery in women, interventions like music, aromatherapy, massage, and virtual reality have been shown to reduce preoperative anxiety and postoperative pain. These interventions demonstrate cost-effectiveness, minimal invasiveness, and a low risk of side effects. However, this study does not determine which of these measures is the most effective. Music, in particular, does not require significant manpower or material resources, incurs lower costs, and is therefore recommended as the most favorable intervention. [19-21]

However, there is no relevant systematic review or meta-analysis to evaluate whether anxiety symptoms in patients before gynecological surgery and postoperative pain can be improved through music intervention. Therefore, we searched for relevant studies to evaluate whether music intervention can alleviate preoperative anxiety and postoperative pain.

Discussion should be improved and more explanation and more explanation of current literature should be added. Current discussion is very short.

4. Revise:**Discussion**

In this paper, we analyzed the effects of music on preoperative anxiety and postoperative pain in patients in gynecological surgery. This meta-analysis indicates that patients who listened to music before surgery had significantly improved preoperative anxiety compared to the control group.This meta-analysis also indicates that patients who listened to music after surgery had significant relief in postoperative pain. Moreover, there was a significant improvement in anxiety symptoms after listening to music compared to before. In summary, we should listen to music for patients before and after gynecological surgery.Listening to music can alleviate preoperative anxiety and postoperative pain in patients undergoing gynecological surgery.

Music is one of the most commonly used non-pharmacological interventions to reduce anxiety. Music is a way for people to relax.[31] It helps patients overcome emotional and physical alienation, providing comfort and familiarity in an improved environment, and serves as a pleasant distraction from pain and anxiety. [32]Music significantly reduces preoperative patients' anxiety, heart rate, and other vital signs. [33]

We analyzed the mechanism by which music affects patient anxiety.Music is a powerful stimulus that evokes and regulates emotions[34]. Studies have shown that the subject's anxiety may be associated with amygdala activity, and music has a significant impact on the amygdala. [36, 37]Music can also have a direct impact on the limbic system of the brain, the reticular structure of the brainstem, and the body's internal organs and functions. For people, harmonious melodies can enhance mental well-being, divert attention, promote relaxation, and reduce tension and anxiety.[38]

Regarding the effect of music on anxiety, other studies have also confirmed the findings of this meta-analysis. In a study on anxiety during cesarean section, researchers found that the anxiety levels of patients in the music group during surgery were lower than those of the control group.[39] Another study also showed that,the use of music-based intervention to reduce postoperative opioid requirements, and music-based intervention may also reduce anxiety, depression, and pain catastrophizing.[40]It was consistent with the results of our study.

In a study on music intervention for dental anxiety patients, it was found that music significantly reduced heart rate, systolic blood pressure fluctuation, diastolic blood pressure fluctuation, anxiety scores, pain scores. Music also significantly decreased anxiety and pain levels while improving patient cooperation. [41]It was consistent with the results of our study.

In a study on music intervention for cataract surgery patients, researchers found that listening to music during cataract surgery significantly reduced self-reported anxiety levels before, during, and after the procedure. Moreover, it led to a significant decrease in postoperative blood pressure. [42]

Another study also indicated that listening to music is associated with a significant reduction in anxiety and pain after cardiac surgery.[43]A meta analysis showed that music therapy can significantly reduce anxiety, pain, HR, SBP, and postoperative opioid use and even improve SpO2 in patients who undergo cardiothoracic surgery. Music therapy has a positive effect on patients after cardiothoracic surgery with few side effects. [44]It was consistent with the results of our study.

Because of the risk of bias in the randomized controlled trials and the inclusion of observational studies in this paper, the quality of evidence was rated as low to medium. This indicates that there is limited confidence in these results, and the actual outcomes may differ from those reported. Therefore, more and better quality randomized controlled trials and adherence to reporting bias control systems are recommended.

The conclusions of these studies are consistent with the findings of this meta-analysis, indicating that music can alleviate preoperative anxiety and postoperative pain in patients. It's totally confirmed that music is an effective, safe, and inexpensive non-pharmacological intervention. It can reduce preoperative anxiety and postoperative pain in patients. This is a good choice for preoperative patients to listen to music before and after surgery. It also plays a supportive role for operating room nurses in patient care.
